# A Study on the Brewing of “Prot-Tea” by Blending Spirulina (Arthrospira platensis) With Green Tea

**DOI:** 10.7759/cureus.54906

**Published:** 2024-02-25

**Authors:** Rajesh Kanna Gopal, Sruthilakshmi Govindaraj

**Affiliations:** 1 Microbiology, Saveetha Dental College and Hospitals, Saveetha Institute of Medical and Technical Sciences, Saveetha University, Chennai, IND; 2 Nutrition, Saveetha Medical College and Hospitals, Saveetha Institute of Medical and Technical Sciences, Saveetha University, Chennai, IND

**Keywords:** protein estimation., dpph antioxidant assay, spirulina powder, green tea, prot-tea

## Abstract

Introduction

Among different blue-green algae, Spirulina (*Arthrospira platensis*) is known for its high protein content and is hence considered a dietary single-cell protein. In recent decades, Spirulina has been one of the nutritive additives in food and beverage products to enhance the nutritional value of food products. The aim of this study was to brew a “Prot-Tea” enriched with antioxidants and protein for nutrition care.

Materials and methods

In combination, both the Spirulina and green tea were brewed together in three different formulations: formulation A: 0.5 g of Spirulina powder and 1.5 g of green tea; formulation B: 1 g each of Spirulina powder and green tea; and formulation C: 1.5 g of Spirulina powder and 0.5 g of green tea. All the formulations were brewed in 100 mL of hot water at 80°C for two minutes. Then, all the formulations were subjected to a 2,2-diphenylpicrylhydrazyl free radical scavenging assay and a quantitative protein estimation assay (Lowry method).

Results

Among the three different formulations, formulation B showed greater antioxidant and protein content. The antioxidant activity of formulation B was directly proportional to the concentration, with an IC_50_ value of 37.98 µL/mL. Similarly, the protein content was also higher in formulation B, with 287.33 µg/100 µL. The total antioxidant in formulation B was 47.61 mg of ascorbic acid equivalent. Concurrently, the total protein content was 229.86 mg in 80 mL of the total volume of Prot-Tea yield.

Conclusion

Based on this study, Prot-Tea is considered a future healthy beverage for nutrition care.

## Introduction

Spirulina (*Arthrospira platensis*) is a blue-green alga that constitutes about 50-70% of the protein content and is rich in essential amino acids, including 2-3% of phenylalanine, leucine, valine, tryptophan, threonine, lysine, isoleucine, and methionine [1,2]. Spirulina (single-cell protein) is prescribed as a cheap vegan source of protein as a nutraceutical agent. Other than protein, Spirulina is also rich in B12 (cyanocobalamin), B9 (folic acid), B6 (pyridoxine), B3 (nicotinamide), B2 (riboflavin), vitamin B1 (thiamine), polyunsaturated fatty acids, vitamin D and E (tocopherol), and vitamin A (carotenoids) [3]. C-phycocyanin from Spirulina is a blue-colored, water-soluble phycobiliprotein and a food-grade natural pigment employed in food industries approved by the US FDA and awarded Spirulina as “Generally Recognized as Safe (GRAS)” [4,5]. Additionally, the United States Pharmacopeia Convention, based on the report of the Dietary Supplements Information Expert Committee, awarded Spirulina a “Class A” on several clinical case reports and released it as safe for human consumption. Therefore, dietary Spirulina is considered a “functional food” with additional benefits [6]. Lafarga et al. proposed that the Spirulina biomass, a “superfood,” can be consumed as a nutritional supplement in powder, capsules, or flakes [7]. Thus, Spirulina biomass was used in protein supplements for athletes, preparation of snack items, dried soup, protein concentrate, milkshakes, pasta, snack bars, ice cream, chocolate milk, biscuits, and sauce [8-14].

Based on the medical documentation, Spirulina showed significant anti-inflammatory activities by hampering histamine release in mast cells and reducing IL-4 levels (32%) in allergic rhinitis. Calcium spirulan, a sulfated polysaccharide in Spirulina, exhibits antiviral activity by inhibiting the replication of several enveloped viruses, including influenza A, HIV-1, herpes simplex virus type 1, measles and mumps virus, and human cytomegalovirus [15]. The Spirulina extract inhibits the glycation of hemoglobin and is suitable for diabetic treatment [16]. The diet enriched with Spirulina enhances the glutathione (GSH) levels in the cerebellar region, suppresses the pro-inflammatory cytokines and malondialdehyde in the brain region, and also induces the vasomotor function of the aorta of aged rats [17,18]. Adding Spirulina enhances the antioxidant properties of sports drinks and improves their nutritional value [19].

Spirulina, with promising health benefits, is commercially sold in capsules as a nutraceutical. Phycocyanin pigment is a major protein in Spirulina with antioxidant and anticancer properties. However, healthcare products from Spirulina would be a good choice for consumption in a more suitable way. Therefore, this study aims to blend Spirulina with green tea to not only enhance the antioxidant properties of tea but also supplement good quality dietary protein in the tea.

## Materials and methods

The study was conducted at Saveetha Dental College and Hospitals, Saveetha Institute of Medical and Technical Sciences, Chennai, India.

The brewing of Prot-Tea

For brewing Prot-Tea, commercial Spirulina powder (*A. platensis*) was purchased from Innoram Biogenics (Chennai, India). Simultaneously, for commercial green tea, Lipton Green Tea, without any flavor, was purchased from DMart (Chennai, India). The reverse osmosis (RO) water used was derived from the RO plant at Saveetha Dental College and Hospitals, Chennai, India.

Three different formulations were used: formulation A, which consists of 0.5 g of Spirulina powder and 1.5 g of green tea; formulation B, which consists of 1 g each of both Spirulina powder and green tea; and formulation C, which consists of 1.5 g and 0.5 g of Spirulina powder and green tea, respectively. To each formulation, 100 mL of hot water (70-80°C) was added and incubated for one minute. Then, the tea extracts were filtered and subjected to antioxidant assays and protein estimation. The best formulation will have the maximum amount of antioxidant and protein content.

Antioxidant assay

About 0.135 mM of 2,2-diphenylpicrylhydrazyl (DPPH) solution was prepared in methanol. Different volumes of Prot-Tea formulations ranging from 5 to 320 µL/mL and 2.5 mL of the DPPH solution were combined separately. An ultraviolet-visible spectrophotometer (Hitachi U-2900, Hitachi, Ltd., Tokyo, Japan) was used to detect the optical density (OD) values at 517 nm after the mixture had been vortexed and incubated for 30 minutes. Ascorbic acid was used as a reference standard. Distilled water (dis. H_2_O) was used as a blank. The percentage of DPPH inhibition was calculated using the following formula [20]: % DPPH inhibition = [(OD of control - OD of the test sample) / (OD of control)] × 100.

Estimation of protein

For the preparation of the Lowry reagent, 2% sodium carbonate in 0.1 N NaOH (sodium hydroxide) was mixed with 1% sodium potassium tartrate solution in a 0.5% copper sulfate solution that was freshly made for the assay. Equally diluted Folin-Ciocâlteu reagent in dis. H_2_O was taken. Bovine serum albumin was used as the reference standard (1 mg/mL). About 100 uL of Prot-Tea formulations were taken as a test sample. The test samples, or different concentrations of the reference standard (50-1,600 µg/mL), were made up to 1 mL using dis. H_2_O, and 1 mL of dis. H_2_O was used as a blank. A total of 5 mL of Lowry reagent was added to the test samples, reference standards, and blank, and the mixture was left undisturbed at room temperature for 15 minutes. After that, 0.5 mL of Folin-Ciocâlteu reagent was added and again incubated for 30 minutes at room temperature. At 660 nm, absorbance values were measured and recorded. The concentration of protein content in the test samples was calculated using the reference standard values and a standard graph [21].

Statistical analysis

All the experiments were carried out in triplets, and their average mean was chosen for statistical analysis and graphical representations. The standard error bars were added to the graphical representations.

## Results

The brewing of Prot-Tea

**Figure 1:**
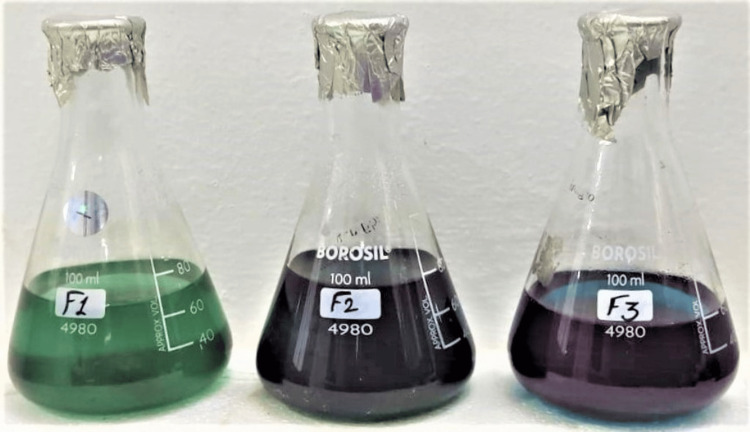
Prot-Tea brewed from the combination of Spirulina powder and green tea F1, formulation 1; F2, formulation 2; F3, formulation 3

Three different Prot-Tea formulations were brewed without adding sugar or other flavors, considering only green tea (antioxidant) and Spirulina powder (protein). Among the three different tea formulations, formulation A (F1) appears green in color, while formulations B (F2) and C (F3) appear in blue-green color (Figure 1).

The total yield of Prot-Tea was 70 mL in formulation A, 80 mL in formulation B, and 60 mL in formulation C. The difference in yield was due to the high green tea and low Spirulina powder in formulation A (70 mL) and the low green tea and high Spirulina powder in formulation C (60 mL). Hence, 80 mL was obtained in formulation B, which had an equal amount of both green tea and Spirulina powder.

Antioxidant assay (DPPH inhibition)

**Figure 2:**
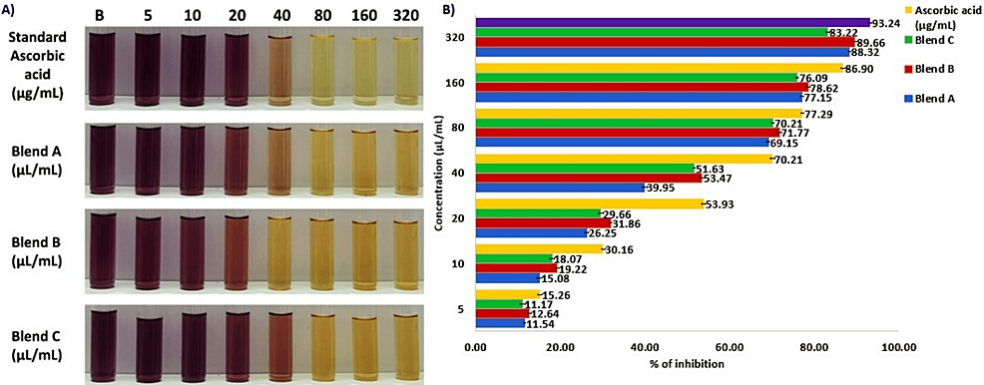
(A) Photograph showing the antioxidant activity (DPPH inhibition) of Prot-Tea formulations (B denotes blank). (B) Graph showing the antioxidant activity (DPPH inhibition) of different Prot-Tea formulations The standard deviation is represented by error bars. DPPH, 2,2-diphenylpicrylhydrazyl

Based on the results in Figure 2A, the purple color indicates the oxidation of DPPH free radicals. However, the pale yellow color indicates the inhibition of DPPH free radicals, showing antioxidant activity. Thus, it is obvious that the antioxidant activity was greater when the concentration of the Prot-Tea formulations increased (concentration is inversely proportional to DPPH free radicals) (Figure 2B). Moreover, among the three different Prot-Tea formulations, formulation B showed greater antioxidant activity (Figure 2).

The IC_50_ values of three Prot-Tea formulations were 46.81 µL/mL, 37.98 µL/mL, and 43.07 µL/mL for formulations A, B, and C, respectively. Hence, comparatively, formulation B was top-ranked with a greater IC_50_ value (i.e., greater antioxidants at a lower concentration). Therefore, the total antioxidant content in formulation B was equivalent to 47.61 mg of ascorbic acid in 80 mL of volume.

Estimation of protein

**Figure 3:**
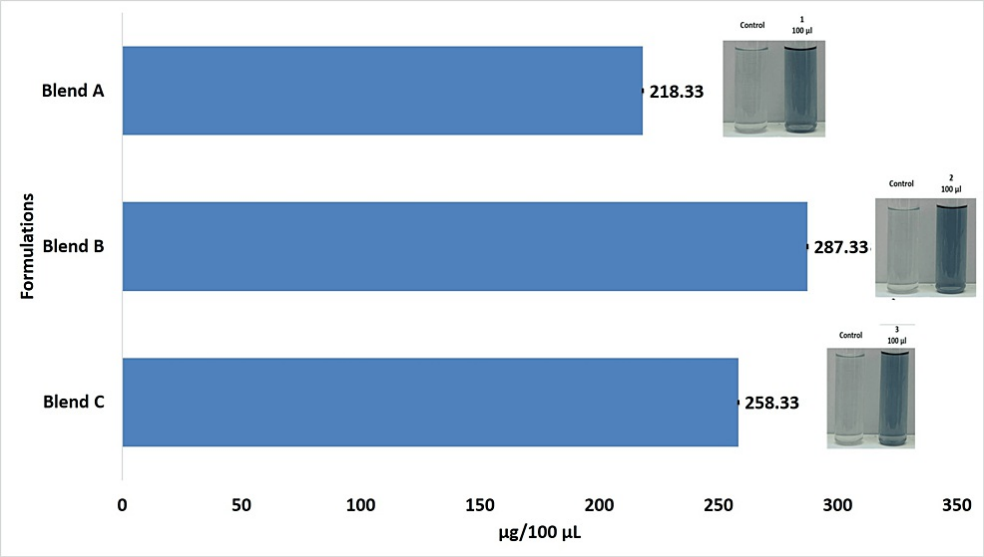
(A) Standard protein (BSA). (B) Graph showing protein content in the three different Prot-Tea formulations The standard deviation is represented by error bars. BSA, bovine serum albumin

Among the three different Prot-Tea formulations, the protein content was found to be greater in formulation B than in formulations A and C. The protein content in formulation B was 287.33 µg/100 µL, and in formulations A and C, it was 218.33 µg/100 µL and 258.33 µg/100 µL, respectively (Figure 3).

The total protein content in formulation A was 152.83 mg in 70 mL of volume. Simultaneously, the total protein content in formulations B and C was 229.86 mg in 80 mL of volume and 155 mg in 60 mL of volume, respectively. Therefore, the total protein content was also higher in formulation B. About 22.98% of the protein was found to be extracted in the Prot-Tea (formulation B).

The three different tea formulations consist of both the antioxidants from green tea and the protein content from Spirulina powder. However, based on the results obtained from total antioxidants and total protein content, formulation B has both greater antioxidants and protein content, with 47.61 mg of ascorbic acid equivalents and 229.86 mg of protein content, than formulations A and C.

## Discussion

The methanol extract of *Arthrospira maxima* showed potential antioxidant activity with an IC_50_ value of 180 µg/mL [22]. However, C-phycocyanin also reported antioxidant activity, with an IC_50_ value of 0.185 mg/mL [23]. In the present study, among three different formulations, formulation B (IC_50_: 37.81 µL/mL) obtained the top rank in the list with a greater amount of antioxidants than A (IC_50_: 43.07 µL/mL) and C (IC_50_: 46.81 µL/mL). Patel et al. found that C-phycocyanin derived from *Lyngbya* had greater inhibition of peroxide free radicals, with an IC_50_ value of 6.63 µM. In comparison, the IC_50_ values for *Spirulina* and *Phormidium* were 12.15 µM and 12.74 µM, respectively [24]. Hence, the antioxidant richness is greater in formulation B due to the effectiveness of extraction than that of A and C. Finally, the estimated total volume of antioxidants in formulation B was equivalent to 47.61 mg of ascorbic acid.

In the current investigation, the total percentage of protein extracted in formulation B was about 22.98%, which was found to be very low in A (15.28%) and C (15.5%). However, approximately 42% of protein recovery was recorded from Spirulina by using methanol and ethanol solvents [25]. In another study, about 28.4% of protein content was derived from 100 g of Spirulina biomass by using an ultrasound mechanism with a protein recovery rate of 50% [26]. Based on a clinical study, the Spirulina extract rectifies the symptoms of allergic rhinitis patients by suppressing IL-4 and Th2 cell differentiation [27].

Direct consumption of Spirulina (4,500 mg) in 16 systemic arterial hypertension patients for 12 weeks along with angiotensin-converting enzyme inhibitors resulted in controlled systolic blood pressure, sE-selectin, sVCAM-1, and endothelin-1 levels, and elevated antioxidant GSH levels [28]. Similarly, the phycocyanin levels in Spirulina had both anti-inflammatory and insulin-sensitizing activities [29]. In another study, 1.5 g of Spirulina was administered to 920 pregnant women from the 28th to the 42nd postnatal day, and a significant increase in hemoglobin levels was found compared to iron and folic acid supplementation [30]. As a result, in the present study, an equal proportion of green tea and Spirulina powder got good results in terms of both the antioxidant levels and dietary protein content in formulation B.

As a result of the study, the resultant formulation, rich in antioxidants and protein content, will be the best choice for Spirulina consumption as a healthy beverage.

Limitation

The allergic reaction of some people to Spirulina is the main limitation of the study.

## Conclusions

Tea is a widely consumed beverage around the world. According to the findings of this study, the combination of green tea and Spirulina provides a modern source of antioxidants and dietary protein, making it a healthy option for healthcare and nutrition.

## References

[1] Soni RA, Sudhakar K, Rana RS (2017). Spirulina - from growth to nutritional product: a review. Trends Food Sci Technol.

[2] da Silva Vaz B, Moreira JB, de Morais M, Costa JA (2016). Microalgae as a new source of bioactive compounds in food supplements. Curr Opin Food Sci.

[3] Mathur M (2018). Bioactive molecules of Spirulina: a food supplement. Bioactive Molecules in Food. Reference Series in Phytochemistry.

[4] Park WS, Kim HJ, Li M (2018). Two classes of pigments, carotenoids and C-phycocyanin, in spirulina powder and their antioxidant activities. Molecules.

[5] Tavanandi HA, Raghavarao KS (2020). Ultrasound-assisted enzymatic extraction of natural food colorant C-phycocyanin from dry biomass of Arthrospira platensis. LWT.

[6] Bortolini DG, Maciel GM, Fernandes IA, Pedro AC, Rubio FT, Branco IG, Haminiuk CW (2022). Functional properties of bioactive compounds from Spirulina spp.: current status and future trends. Food Chem (Oxf).

[7] Lafarga T, Fernández-Sevilla JM, González-López C, Acién-Fernández FG (2020). Spirulina for the food and functional food industries. Food Res Int.

[8] Lucas BF, de Morais MG, Santos TD, Costa JA (2018). Spirulina for snack enrichment: nutritional, physical and sensory evaluations. LWT.

[9] da Silva PC, Toledo T, Brião V, Bertolina TE, Costa JA (2021). Development of extruded snacks enriched by bioactive peptides from microalga Spirulina sp. LEB 18. Food Biosci.

[10] Menegotto AL, de Souza LE, Colla LM (2019). Investigation of techno-functional and physicochemical properties of Spirulina platensis protein concentrate for food enrichment. LWT.

[11] Lucas BF, da Rosa AP, de Carvalho LF, de Morais M, Santos TD, Costa JA (2020). Snack bars enriched with spirulina for schoolchildren nutrition. Food Sci Technol.

[12] da Silva Faresin L, Devos RJ, Reinehr CO, Colla LM (2022). Development of ice cream with reduction of sugar and fat by the addition of inulin, Spirulina platensis or phycocyanin. Int J Gastron Food Sci.

[13] de Oliveira TT, dos Reis IM, de Souza MB (2021). Microencapsulation of Spirulina sp. LEB-18 and its incorporation in chocolate milk: properties and functional potential. LWT.

[14] Almeida LM, da Silva Cruz LF, Machado BA (2021). Effect of the addition of Spirulina sp. biomass on the development and characterization of functional food. Algal Res.

[15] Karkos PD, Leong SC, Karkos CD, Sivaji N, Assimakopoulos DA (2011). Spirulina in clinical practice: evidence-based human applications. Evid Based Complement Alternat Med.

[16] Paramanya A, Farah MA, Al-Anazi KM, Devkota HP, Ali A (2023). Exploring the potential of spirulina (Arthrospira platensis) aqueous extract in preventing glycation of hemoglobin and pBR322 plasmid. Pharmacogn Mag.

[17] Trotta T, Porro C, Cianciulli A, Panaro MA (2022). Beneficial effects of spirulina consumption on brain health. Nutrients.

[18] Majewski M, Klett-Mingo M, Verdasco-Martín CM, Otero C, Ferrer M (2022). Spirulina extract improves age-induced vascular dysfunction. Pharm Biol.

[19] Sadeghi T, Marvizadeh MM, Ebrahimi F (20221022034202219536801516). Assessment of nutritional and antioxidant activity of sport drink enriched with Spirulina platensis. J Chem Health Risks.

[20] Blois MS (1958). Antioxidant determinations by the use of a stable free radical. Nature.

[21] Lowry OH, Rosebrough NJ, Farr AL, Randall RJ (1951). Protein measurement with the Folin-phenol reagent. J Biol Chem.

[22] Miranda MS, Cintra RG, Barros SB, Mancini Filho J (1998). Antioxidant activity of the microalga Spirulina maxima. Braz J Med Biol Res.

[23] Renugadevi K, Valli Nachiyar C, Sowmiya P (2018). Antioxidant activity of phycocyanin pigment extracted from marine filamentous cyanobacteria Geitlerinema sp TRV57. Biocatal Agric Biotechnol.

[24] Patel A, Mishra S, Ghosh PK (2006). Antioxidant potential of C-phycocyanin isolated from cyanobacterial species Lyngbya, Phormidium and Spirulina spp. Indian J Biochem Biophys.

[25] Sela K, Budhijanto W, Budiman A (2021). Protein extraction from Spirulina platensis by using ultrasound assisted extraction: effect of solvent types and extraction time. Key Eng Mater.

[26] Vernès L, Abert-Vian M, El Maâtaoui M, Tao Y, Bornard I, Chemat F (2019). Application of ultrasound for green extraction of proteins from spirulina. Mechanism, optimization, modeling, and industrial prospects. Ultrason Sonochem.

[27] Mao TK, Van de Water J, Gershwin ME (2005). Effects of a Spirulina-based dietary supplement on cytokine production from allergic rhinitis patients. J Med Food.

[28] Martínez-Sámano J, Torres-Montes de Oca A, Luqueño-Bocardo OI, Torres-Durán PV, Juárez-Oropeza MA (2018). Spirulina maxima decreases endothelial damage and oxidative stress indicators in patients with systemic arterial hypertension: results from exploratory controlled clinical trial. Mar Drugs.

[29] Zheng H, Powell JE, Steele MI, Dietrich C, Moran NA (2017). Honeybee gut microbiota promotes host weight gain via bacterial metabolism and hormonal signaling. Proc Natl Acad Sci U S A.

[30] Niang K, Ndiaye P, Faye A (2017). Spirulina supplementation in pregnant women in the dakar region (Senegal). Open J Obstet Gynecol.

